# Keeping the Home Fires Burning Cleaner: Solid Fuel Use, Health, and the Millennium Development Goals

**DOI:** 10.1289/ehp.114-a178

**Published:** 2006-03

**Authors:** Scott Fields

Many people in industrialized nations give little thought to central heating, electric lighting, and flick-of-a-switch cooking. But more than half of the people in the world rely on solid fuels to heat and light their homes and cook their food. After assessing global solid fuel use, researchers estimate that 52% of the world’s people burn solid fuels such as wood, coal, peat, and dung **[*EHP*** **114:373–378; Rehfuess et al.]**. Burning these fuels, they say, can profoundly harm the health of the people exposed to them as well as damage regional environments.

The researchers set out to assess household solid fuel use on a country-by-country basis. In this report the researchers describe the impact that increases in worldwide dependence on solid fuels would have on meeting the UN Millennium Development Goals. These eight goals set in 2000 aim to reduce poverty, hunger, disease, illiteracy, environmental degradation, child mortality, and gender inequality, and improve maternal health.

For 52 wealthier countries (in which per-capita income is more than US$10,500), the researchers assumed that fewer than 5% of the population depended on household solid fuels. For 147 poorer countries, the researchers melded surveys and modeling where possible. They collected national census or household survey data on solid fuel use—often for cooking only, the fate of most household solid fuel—for 93 countries. For 36 countries that had no such data available, the researchers modeled solid fuel use based on factors such as gross national income and each country’s proportion of rural dwellers. Finally, for 18 countries, many of them small island states such as the Cook Islands, the Maldives, and Tuvalu, there were not enough data available to feed the models. These 18 countries were excluded from the study.

Solid fuel use varied widely among the low-income regions, from 77% in sub-Saharan Africa to 16% in Latin America. According to the authors, 3.2 billion people depended on solid fuels as of 2003, not many fewer than the estimated 3.4 billion using such fuels three years earlier. About 75% of these people burned biomass fuels, which can lead to depletion of natural resources when harvested, and which typically are burned in crude stoves or open fires, resulting in incomplete combustion and releasing high levels of greenhouse gases.

Significantly reducing global dependence on solid fuels is necessary if the Millennium Development Goals—in particular reducing child mortality and improving maternal health—are to be met, the researchers explain. Burning solid fuels in cooking rooms, where women and their children typically spend much of their time, fills homes with pollutants such as carbon monoxide, particulate matter, and other carcinogens that penetrate deep into the lungs.

The researchers believe global society must embrace safe alternatives to solid fuels if the Millennium Development Goals are to be achieved, and they point to examples of interventions already in place that have rapidly bettered the lot of solid fuel users. It is essential, they write, that nations work together to make the necessary policy changes and implement technical solutions.

## Figures and Tables

**Figure:**
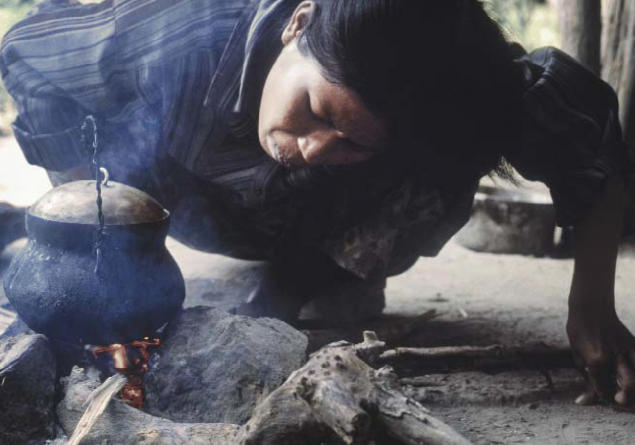
Unhealthy home fires. An assessment of solid fuel use reveals that continued widespread global dependence on such fuels for household needs will impede success in meeting the UN Millennium Development Goals.

